# Microtubules Modulate F-actin Dynamics during Neuronal Polarization

**DOI:** 10.1038/s41598-017-09832-8

**Published:** 2017-08-29

**Authors:** Bing Zhao, Durga Praveen Meka, Robin Scharrenberg, Theresa König, Birgit Schwanke, Oliver Kobler, Sabine Windhorst, Michael R. Kreutz, Marina Mikhaylova, Froylan Calderon de Anda

**Affiliations:** 1 RG Neuronal Development, Center for Molecular Neurobiology Hamburg (ZMNH), University Medical Center Hamburg-Eppendorf, 20251 Hamburg, Germany; 2 Emmy-Noether Group “Neuronal Protein Transport”, Center for Molecular Neurobiology (ZMNH), University Medical Center Hamburg-Eppendorf, 20251 Hamburg, Germany; 3 RG Neuroplasticity, Leibniz Institute for Neurobiology, 39118 Magdeburg, Germany; 4 Leibniz Guest Group “Dendritic Organelles and Synaptic Function”, Center for Molecular Neurobiology (ZMNH), University Medical Center Hamburg-Eppendorf, 20251 Hamburg, Germany; 5 Department of Biochemistry and Signal Transduction, University Medical Center Hamburg-Eppendorf, 20246 Hamburg, Germany; 6 Combinatorial Neuroimaging Core Facility (CNI), Leibniz Institute for Neurobiology, 39118 Magdeburg, Germany

## Abstract

Neuronal polarization is reflected by different dynamics of microtubule and filamentous actin (F-actin). Axonal microtubules are more stable than those in the remaining neurites, while dynamics of F-actin in axonal growth cones clearly exceed those in their dendritic counterparts. However, whether a functional interplay exists between the microtubule network and F-actin dynamics in growing axons and whether this interplay is instrumental for breaking cellular symmetry is currently unknown. Here, we show that an increment on microtubule stability or number of microtubules is associated with increased F-actin dynamics. Moreover, we show that Drebrin E, an F-actin and microtubule plus-end binding protein, mediates this cross talk. Drebrin E segregates preferentially to growth cones with a higher F-actin treadmilling rate, where more microtubule plus-ends are found. Interruption of the interaction of Drebrin E with microtubules decreases F-actin dynamics and arrests neuronal polarization. Collectively the data show that microtubules modulate F-actin dynamics for initial axon extension during neuronal development.

## Introduction

Axon formation is a hallmark of neuronal polarization in early developing hippocampal and cortical pyramidal neurons^1–5^. Neurons initially extend several neurites (Stage 2;^1^), from which usually those with the fastest growth rate become axons (Stage 3;^1^), while the remaining neurites transform into dendrites^1, 6^. However, our understanding of axon selection is still far from being complete. It has been shown that microtubule stabilization in the axonal shaft precedes the elongation and specification of the axon^7–9^, whereas global microtubule stabilization induces the formation of multiple axons^10^. In addition, it has been demonstrated that neuronal polarization or axon formation could occur through cell-length-dependent accumulation of microtubules without selective microtubule stabilization^11^. On the other hand, F-actin is more dynamic within axonal as compared to dendritic growth cones and the F-actin depolymerizing agent cytochalasin D causes neurons to develop multiple axons^12^. Along these lines several signaling mechanisms have been shown to regulate extensive remodeling of the cytoskeleton, which in turn precedes and instructs axon growth^7–9, 13^. However, whether the interplay between microtubules and F-actin sets the conditions for axon selection and elongation is still not well understood. Several lines of evidence show that axon selection can be induced by extracellular cues in a stochastic manner^3, 14–16^, suggesting that F-actin instability might lead to eventual microtubule stabilization. Other reports indicate that centrosome and Golgi apparatus positioning can predict axon selection^2, 17–21^, indirectly suggesting that microtubules might play a modulating role. Consequently, it is possible that microtubules might determine F-actin dynamics prior to and during axon formation to set up the conditions for breaking cellular symmetry.

It has been recently reported that Drebrin promotes microtubule entry into spines of mature neurons, which are F-actin rich structures^22^. Drebrin inhibits cofilin-induced severing of F-actin and stabilizes F-actin^23, 24^. Drebrin also binds EB3 to promote neurite formation^25^. A recent study provides evidence that Drebrin contributes to the coordination of the actin and microtubule cytoskeleton during the initial stages of axon branching^26^. Drebrin is therefore a suitable candidate for investigating the molecular cross-talk between microtubule and actin prior and during axon extension. To address this important question we characterized the interplay between microtubule and F-actin dynamics in developing neurons during neuronal polarization.

## Results

### Drebrin E is segregated to growth cones with higher F-actin treadmilling rate prior and during axon extension

**Figure 1:**
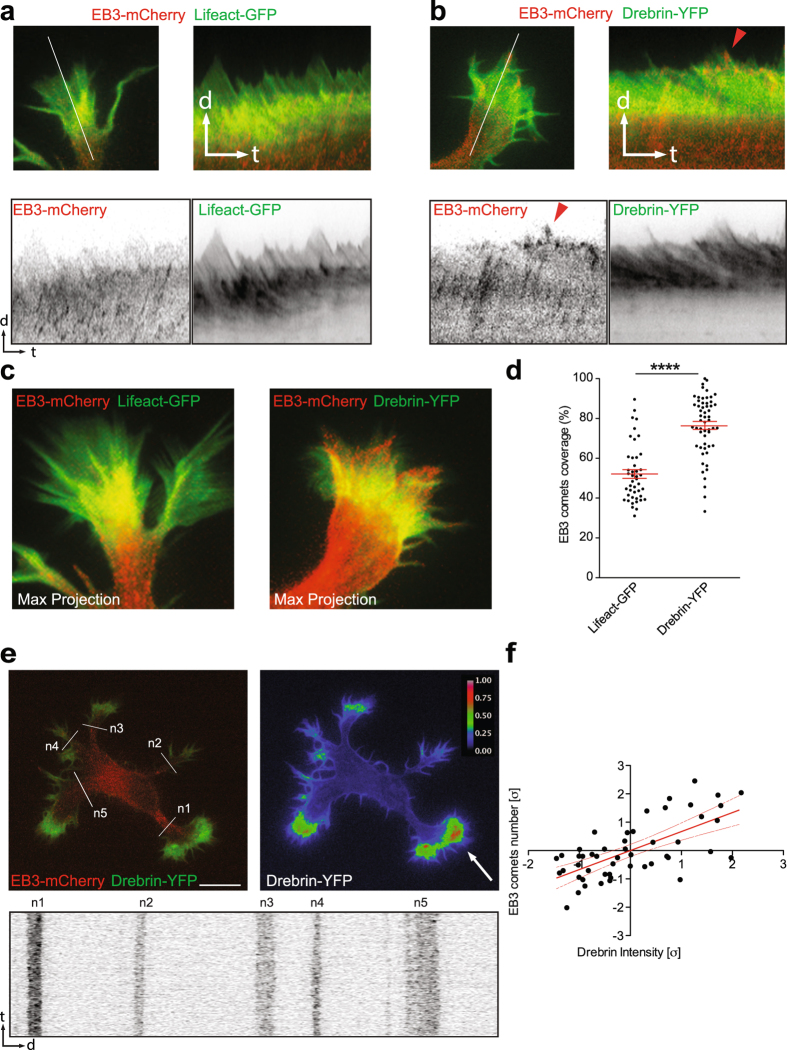
Drebrin promotes microtubule entry into growth cones. (**a–c**) Drebrin-YFP expression promotes entry of EB3 comets to the peripheral domain of growth cones compared to growth cones expressing Lifeact-GFP (kymographs from white line). (**d**) Quantification of growth cone area occupied by EB3 comets. In percentage (%), EB3-mCherry/Lifeact-GFP cells = 52.12 ± 2.182; n = 10 cells from at least three different cultures; EB3-mCherry/Drebrin-YFP cells = 76.51 ± 2.019; n = 10 cells from at least three different cultures; ****p < 0.0001 by t test; Mean ± SEM (**e**) Representative stage 2 cell transfected with Drebrin-YFP and EB3-mCherry showing the neurite with higher Drebrin intensity receives more EB3 comets. The neurite (n1) that is enriched with overexpressed Drebrin (white arrow) received higher number of EB3 comets (see the kymograph from white lines, n1–n5) (**f**) Pearson correlation analysis of Drebrin-YFP intensity and EB3 comets number entering into the neurites (per 5 min) from stage 2 cells. n = 8 cells from at least three different cultures; Values were normalized according to standard score and axes are represented in units of standard deviation [σ]. Line equation: Y = 0.6698*X + 5.742e-008; Pearson r = 0.6698, p < 0.0001. Dashed lines represent 95% confidence intervals. Scale bar: 10 μm (**e**).

**Figure 2:**
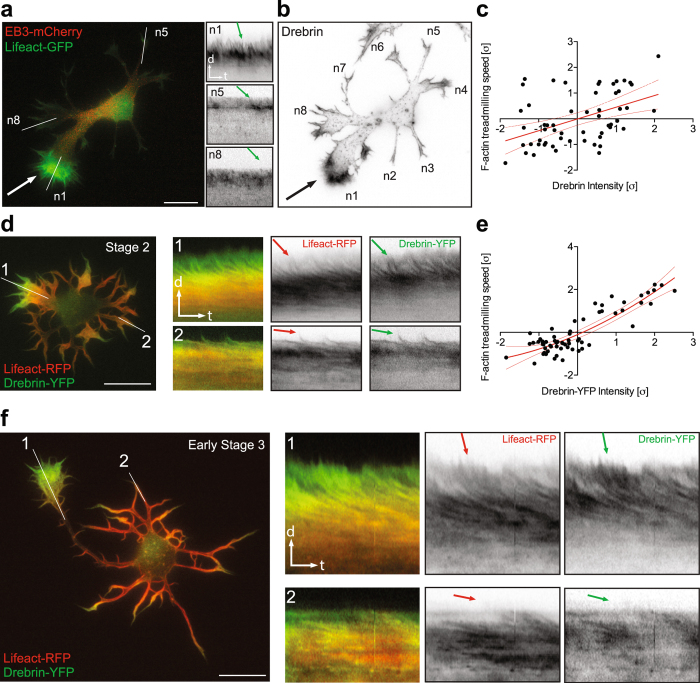
Drebrin is segregated to growth cones with higher F-actin treadmilling rates. (**a**,**b**) Endogenous Drebrin is enriched in growth cones with higher treadmilling rates. The neurite (white arrow) that has faster - F-actin treadmilling at the growth cone (kymographs n1) accumulates more Drebrin (black arrow in **b**). (**c**) Pearson correlation analysis of endogenous Drebrin intensity and F-actin treadmilling speed in growth cones from stage 2 cells. n = 10 cells from at least three different cultures; Values were normalized according to standard score and axes are represented in units of standard deviation [σ]. Line equation: Y = 0.4560*X − 0.009099; Pearson r = 0.4549, p < 0.0004. Dashed lines represent 95% confidence intervals. (**d**) Stage 2 Drebrin-overexpressed cell segregates preferentially Drebrin-YFP to the growth cones with faster treadmilling (kymographs from white line 1, 2). (**e**) Second order polynomial fit of Drebrin-YFP intensity and F-actin treadmilling speed in growth cones from stage 2 cells. n = 11 cells from at least three different cultures. Values were normalized according to standard score and axes are represented in units of standard deviation [σ]. Line equation: Y = 0.8723*X − 1.270e-007; R squared value is 0.7740. Dashed lines represent 95% confidence intervals. (**f**) Early stage 3 Drebrin-overexpressed cell segregates Drebrin-YFP preferentially to the future axon with a more actin-dynamic growth cone (kymographs from white line 1, 2). Scale bar: 10 μm (**a**,**d**,**f**).

**Figure 3:**
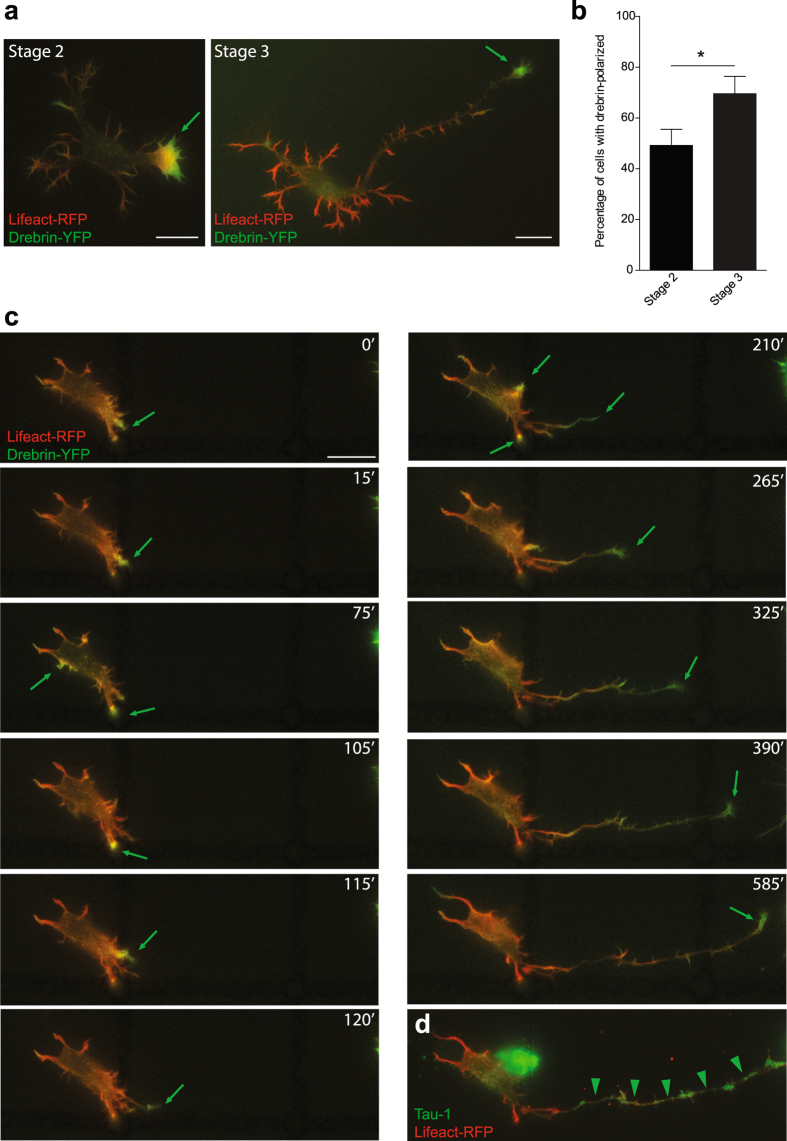
Drebrin localization predicts the position of axon outgrowth. (**a**) Stage 2 and stage 3 neurons transfected with Drebrin-YFP and Lifeact-RFP showing polarized Drebrin signal at one neurite tip. (**b**) Quantifications showing percentage of stage 2 and stage 3 cells with polarized Drebrin signal. In percentage %, stage 2 = 49.21 ± 6.349, stage 3 = 69.57 ± 6.859, stage 2 cells n = 63, stage 3 cells n = 46, p = 0.0337, Student’s t test, *p < 0.05; Mean ± SEM. (**c**) Neuron expressing Drebrin-YFP and Lifeact-RFP forms an axon from the dynamic neurite, which shows more Drebrin-YFP (**d**) Tau-1 staining confirms the axonal identity of the cell. Scale bar: 10 μm (**a** and **c**).

We decided to study the impact of Drebrin overexpression on microtubule and F-actin dynamics directly. Rat hippocampal neurons were transfected with Lifeact-GFP or Drebrin-YFP together with the microtubule plus-end marker EB3-mCherry before plating. 24 hrs later, developing neurons (stage 2 to early stage 3) were imaged for 5 min with a frame rate of 2 sec. Drebrin-YFP overexpression promoted the entry of EB3-mCherry to the peripheral domain of growth cones (Fig. 1a–d, Video 1). However, EB3 rarely went further than the central domain of the growth cone when neurons co-expressed EB3-mCherry and Lifeact-GFP. This was evidenced by quantification of the percentage of EB3 comets coverage performed in Lifeact-GFP and Drebrin-YFP expressing cells. (Fig. 1a–d, Video 1). It has been previously shown that endogenous Drebrin localized in the transitional domain of growth cones^25, 27^. However, we found that Drebrin-YFP localized in the peripheral as well as in the transitional domain of growth cones. Therefore, we analyzed the localization of endogenous Drebrin. We found that Drebrin is predominantly localized in the transitional domain in some growth cones; nevertheless, it is not precluded from the growth cone periphery (Supplementary Figure 1a,b). This confirms a similar distribution of endogenous and overexpressed Drebrin signal. In addition, we found that endogenous Cofilin is preferentially enriched along with endogenous Drebrin or overexpressed Drebrin-YFP in growth cones (Supplementary Figure 1c–h). Next, we determined the Drebrin-YFP signal intensity and number of EB3 comets entering growth cones of stage 2 cells. The quantification shows a correlation between the number of EB3 comets and the intensity of Drebrin signal; growth cones that received more EB3 comets had more Drebrin-YFP signal (Fig. 1e,f). Remarkably, we also found that the endogenous Drebrin in cultured neurons is enriched in the growth cone with a higher actin treadmilling rate (Fig. 2a–c; Video 2). This result in principle seems counterintuitive given the fact that Drebrin stabilizes F-actin^24^. However, higher Cofilin levels in endogenous Drebrin enriched growth cones (Supplementary Figure 1c,d,g and h) might lead to a relatively higher F-actin treadmilling rate at these sites. We next decided to examine via live-imaging microscopy whether Drebrin segregates to the growth cones with a higher treadmilling rate. To this end, rat hippocampal neurons were transfected with Drebrin-YFP together with Lifeact-RFP before plating. 24 hrs after plating, stage 2 and early stage 3 cells were imaged via time-lapse microscopy. We observed that Drebrin-YFP is preferentially present in growth cones with a higher treadmilling rate in stage 2 cells (Fig. 2d,e; control cell of Video 10) and axonal growth cones with higher treadmilling rates in early stage 3 cells (Fig. 2f; Video 3). Finally, we performed long-term live imaging experiments (12–20 hrs) to track the axon formation. We observed that in around 50% of stage 2 cells Drebrin-YFP is enriched in only one neurite and in approximately 70% of stage 3 cells it is enriched in the growing axon (Fig. 3a,b). Accordingly, in long-term live imaging, we found that Drebrin-YFP at stage 2 is changing position among different growth cones over time. Eventually, Drebrin-YFP stabilizes in one place, where the axon emerges (Fig. 3c,d; Video 4). These results suggest a molecular pathway, which promotes increased F-actin dynamics in growth cones and hence axon formation. However, the F-actin stabilizing role of Drebrin is well documented^24^ and our results show that in Drebrin expressing cells, the overall F-actin treadmilling speed is decreased compared to cells expressing just Lifeact (Supplementary Figure 2). Although, overall F-actin dynamics are decreased in Drebrin over-expressing cells, the growth cone of the axon still shows faster F-actin treadmilling than that of the minor processes (Supplementary Figure 2). Furthermore, the presence of more Cofilin in overexpressed Drebrin enriched growth cones (Supplementary Figure 1e–h) might contribute to a higher F-actin treadmilling rate at these sites. Our data show a positive correlation between number of EB3 comets (growing microtubules) and Drebrin accumulation in growth cones. In addition, it has been reported that axon formation occurs through accumulation of microtubules^11^. Therefore we hypothesized that the limiting factor behind increased F-actin dynamics during axon extension is the specific segregation/stabilization of microtubules towards the axonal growth cone.

### Microtubule dynamics affect F-actin dynamics

**Figure 4:**
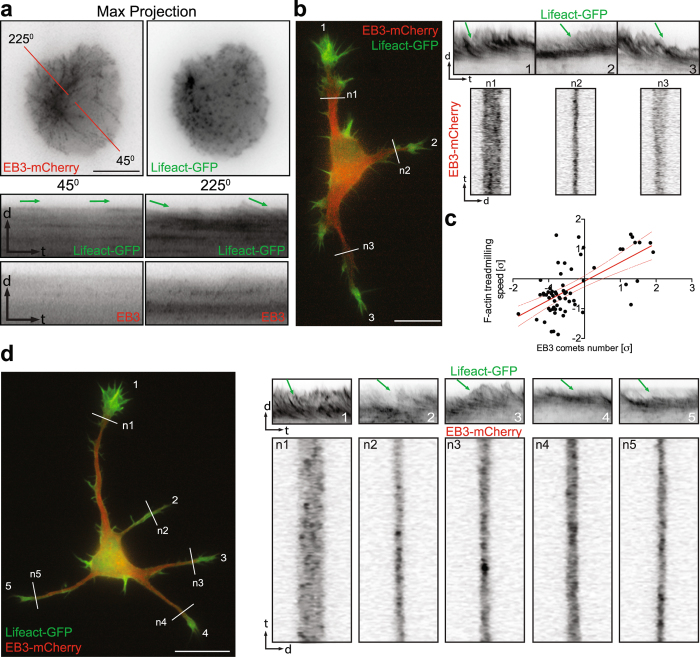
Microtubule dynamics predict F-actin treadmilling. (**a**) F-actin initiated treadmilling in places where more EB3 comets arrived (kymograph at 225**°**). In places where few EB3 comets were detected F-actin treadmilling is absent (kymograph at 45°). (**b)** Stage 2 cell shows that the neurite with more EB3 comets (kymographs from white lines) has faster F-actin treadmilling (kymographs from 1–3). (**c**) Pearson correlation analysis of number of EB3 comets and F-actin treadmilling speed in growth cones from stage 2 cells. n = 9 cells from at least three different cultures. Values were normalized according to standard score and axes are represented in units of standard deviation [σ]. Line equation: Y = 0.6337*X − 0.08005; Pearson r = 0.6395, p < 0.0001. Dashed lines represent 95% confidence intervals. (**d**) The longest neurite (n1) of the early stage 3 cell that receives more EB3 comets (kymographs from white lines n1–n5) has faster F-actin treadmilling (kymographs from 1–5). Scale bar: 5 μm (**a**), 10 μm (**b** and **d**).

To gain insight into the microtubule-F-actin interplay during neuronal polarization, in the first set of experiments we studied microtubule and F-actin dynamics using time-lapse microscopy. Rat hippocampal neurons were transfected with Lifeact-GFP together with the microtubule plus-end marker EB3-mCherry before plating. 24 hrs later developing neurons (stage 1 to early stage 3) were imaged for 5 min with a frame rate of 2 sec. In freshly plated cells, which have not shown signs of neurite formation, a low number of EB3 comets correlated with absence of F-actin treadmilling in the cell cortex (Fig. 4a). Hence F-actin initiated treadmilling (typical for growth cone dynamics) was more prominent in regions with an elevated number of EB3 comets (Fig. 4a). Therefore, we determined the number of EB3 comets and the F-actin treadmilling rate in growth cones of stage 2 cells. The quantification shows a correlation between the number of EB3 comets entering the growth cone and the speed of F-actin treadmilling: growth cones with more EB3 comets had a higher F-actin treadmilling rate (Fig. 4b–d; Video 5). It has been previously shown that during axon differentiation virtually no change occurs in average microtubule length^28^. Therefore, elongation of existing microtubules cannot account for the major expansion of the microtubule arrays, as a minor process becomes an axon^28^. In contrast, the number of microtubules increases as a minor process differentiates and grows into an axon^28^. Thus, we hypothesized that more and stable microtubules might increase F-actin dynamics by affecting treadmilling in growth cones.

**Figure 5:**
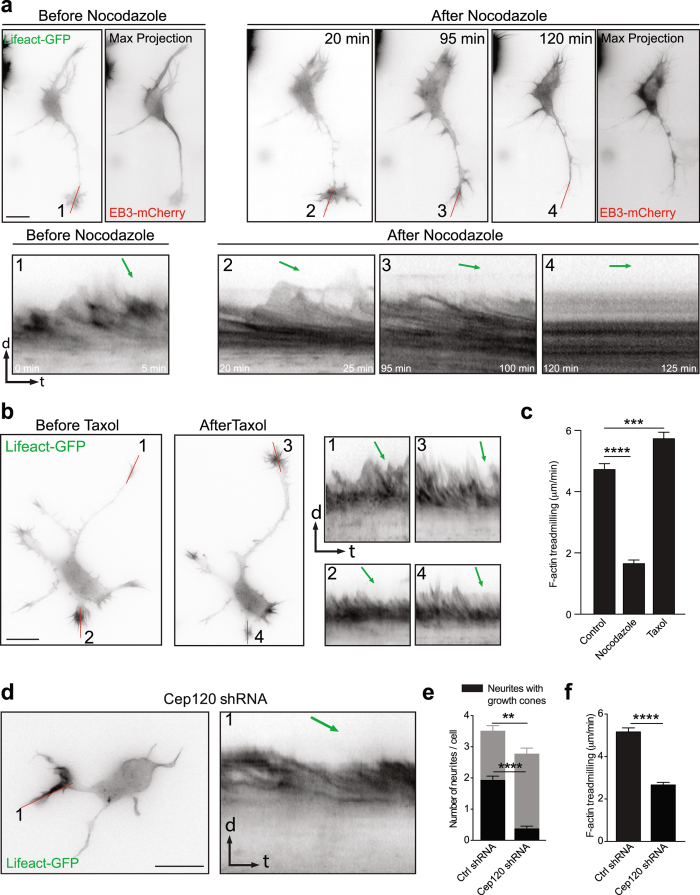
Microtubule dynamics and stability affect growth cone F-actin treadmilling. (**a**) Neuron transfected with Lifeact-GFP and EB3-mCherry before and after nocodazole treatment. The max projection images show EB3-mCherry signal before and after nocodazole treatment (120 min). Kymographs (from red line 1–4) show progressive reduction of F-actin treadmilling after nocodazole treatment. (**b**) Neuron expressing Lifeact-GFP before and after taxol treatment. Taxol treatment increased the F-actin dynamics at growth cones (kymographs 1–4 from red lines 1–4). (**c**) Quantification of F-actin treadmilling in growth cones before and after nocodazole and taxol treatment. Control = 4.7258 ± 0.1918 μm/min; n = 10 cells from at least three different cultures; after nocodazole = 1.6522 ± 0.1183 μm/min; n = 10 cells from at least three different cultures; p < 0.0001 by one-way ANOVA, *post hoc* Dunnett test ****p < 0.0001; after taxol = 5.7273 ± 0.2150 μm/min; n = 10 cells from at least three different cultures; p < 0.0001 by one-way ANOVA, *post hoc* Dunnett test ***p < 0.001**;** Mean ± SEM. (**d**) Transfection of mouse cortical neurons via in utero electroporation with Cep120 shRNA decreased F-actin treadmilling in growth cones, kymograph drawn from red line 1 and green arrow on the kymograph indicates the F-actin treadmilling slope. (**e**) Quantification of total neurites, neurites with and without growth cones, from stage 2 transfected with Control shRNA and Cep120 shRNA were shown. Separate statistical comparisons were made to analyze the differences in total neurite number (black + grey bars) and neurites with growth cones (black bars) among the groups. Neurites per cell: Ctrl shRNA = 3.500 ± 0.1593 VS Cep120 shRNA = 2.767 ± 0.1492, **p = 0.0014 by t test, Mean ± SEM. Growth cones per cells: Ctrl shRNA = 1.923 ± 0.1350 VS Cep120 shRNA = 0.3667 ± 0.08949; n = 26 cells for Ctrl shRNA and n = 30 cells for Cep120 shRNA obtained from at least three different cultures; ****p < 0.0001 by t test, Mean ± SEM. (**f**) Quantification of F-actin treadmilling in growth cones from cells transfected with Control and Cep120-shRNA. Control shRNA = 5.157 ± 0.1927 µm/min, Cep120 shRNA = 2.661 ± 0.1226 µm/min; n = 11 cells per condition, obtained from at least three different cultures; ****p < 0.0001 by t test; Mean ± SEM. Scale bar: 10 μm (**a, b** and **c**).

In the next set of experiments, we addressed the effect of microtubule stability on actin dynamics more directly. We treated stage 2 and early stage 3 hippocampal primary neurons with nocodazole and taxol (microtubule destabilization and stabilizing drugs, respectively) and analyzed their effect on F-actin dynamics in growth cones. Compared to untreated cells, in nocodazole treated cells the F-actin treadmilling in growth cones was significantly reduced (Fig. 5a,c; Video 6). In contrast, in the presence of taxol, which has been shown to promote microtubule advancement into growth cones^10^, F-actin treadmilling was increased (Fig. 5b,c; Video 7). To further investigate this possibility, we down-regulated Cep120, a centrosomal protein, which has been shown to affect microtubule stability^2, 29^. We employed a shRNA knockdown strategy to down-regulate the expression of the centrosomal protein Cep120 early in development. The centrosomal protein Cep120 has been previously shown to control the size of the astral microtubule structure, which couples the centrosome and the nucleus in neuronal progenitors^29^. In addition, it has been shown that Cep120 controls microtubule stability in developing neurons^2^. To investigate the effects of Cep120 down-regulation in early neuronal development, we used a Cep120 shRNA construct for specifically silencing Cep120 expression in cortical neurons and neuronal progenitors^2, 29^. We introduced Cep120 shRNA or control shRNA plasmids together with Lifeact-GFP expressing plasmids in mice brain cortices at embryonic day 15 (E15) and isolated cortical neurons at E17. Neurons were cultured *in vitro* for an additional 24 hrs and time-lapse experiments were performed in stage 2 neurons (5 min, imaging every 2 sec). Cep120 down-regulation affected neuronal development in cultured neurons inducing less and shorter neurites with fewer growth cones as reported previously^2^ (Fig. 5d,e). In previous work we found that down-regulation of Cep120 in the developing cortex disrupts neuronal polarization and precludes axon formation^2^. Furthermore, neuronal migration is impaired^2^. In extension of these findings, we now observed that Cep120 down-regulation significantly decreased F-actin treadmilling speed in neurite tips (Fig. 5f). Taken together these experiments point out an important role of microtubules in controlling F-actin dynamics in the growth cones of developing neurons.

### Disruption of microtubule/Drebrin interaction affects F-actin dynamics and neuronal development

Previous reports demonstrate that Drebrin is a molecular hinge between microtubule and F-actin. Disturbance of the interaction between Drebrin and microtubule should therefore affect F-actin dynamics and ultimately neuronal polarization.

**Figure 6:**
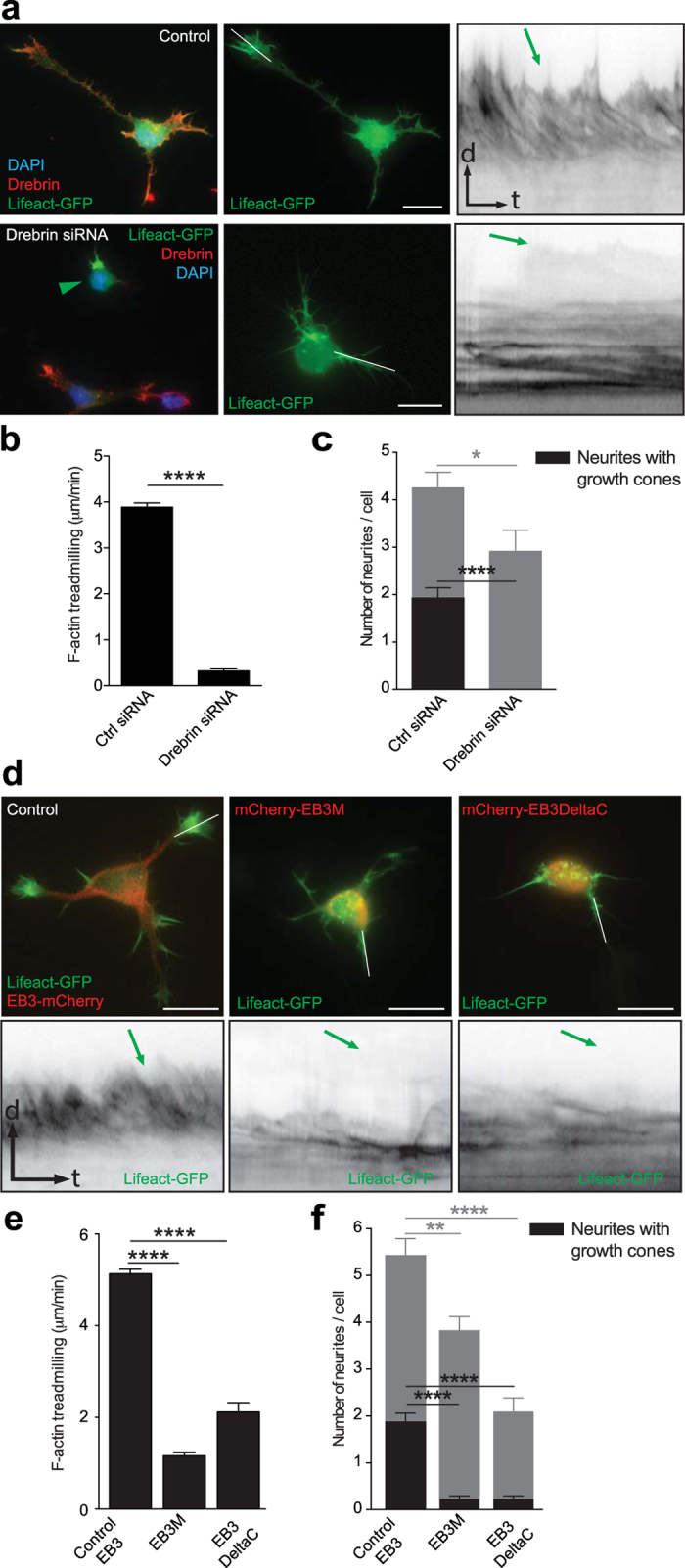
Drebrin knockdown or disruption of microtubule/Drebrin interaction affects F-actin treadmilling and growth cone formation. (**a**) Drebrin siRNA transfection in rat hippocampal neurons decreased F-actin dynamics in growth cones. Drebrin immunostaining confirms efficient knockdown in Drebrin siRNA transfected neuron pointed with green arrowhead. (**b**) Quantification of F-actin treadmilling in growth cones from cells transfected with Control siRNA and Drebrin siRNA. Control siRNA = 3.886 ± 0.0922 μm/min; n = 10 cells from at least three different cultures; Drebrin siRNA = 0.321 ± 0.0630 μm/min; n = 10 cells from at least three different cultures; ****p < 0.0001 by t test; Mean ± SEM. (**c**) Quantification of total neurites, neurites with and without growth cones (gc) from stage 2 and 3 cells transfected with Control siRNA, Drebrin siRNA were shown. Separate statistical comparisons were made to analyze the differences in total neurite number (black + grey bars) and neurites with growth cones (black bars) among the groups. Neurites per cell: Ctrl siRNA = 4.250 ± 0.3286 VS Drebrin siRNA = 2.900 ± 0.4583, Ctrl siRNA n = 12, Drebrin siRNA n = 10 cells from at least three different cultures; *p = 0.0237 by t test. Growth cones per cells: Ctrl siRNA = 1.917 ± 0.2289 VS Drebrin siRNA = 0, Ctrl siRNA n = 12, Drebrin siRNA n = 10 cells from at least three different cultures; ****p < 0.0001 by t test; Mean ± SEM. (**d**) Expression of EB3 truncation mutants (EB3M and EB3DeltaC) decreased F-actin dynamics in growth cones (kymographs from white lines). (**e**) Quantification of F-actin treadmilling in growth cones from cells expressing EB3, EB3M, and EB3DeltaC. Control = 5.130 ± 0.1017μm/min; n = 15 cells from at least three different cultures; EB3M = 1.1623 ± 0.0737μm/min; n = 12 cells from at least three different cultures; p < 0.0001 by one-way ANOVA, *post hoc* Dunnett’s test ****p < 0.0001; EB3DeltaC = 2.1153 ± 0.2027 μm/min; n = 9 cells from at least three different cultures; p < 0.0001 by one-way ANOVA, *post hoc* Dunnett’s test ****p < 0.0001; Mean ± SEM. (**f**) Quantification of total neurites, neurites with and without growth cones (gc) from stage 2 and 3 cells transfected with EB3, EB3M, EB3DeltaC were shown. Separate statistical comparisons were made to analyze the differences in total neurite number (black + grey bars) and neurites with growth cones (black bars) among the groups. Neurites per cell, Control EB3 = 5.414 ± 0.3274 VS EB3M = 3.810 ± 0.3128 or EB3DeltaC = 2.081 ± 0.2986; Control EB3 n = 29, EB3M n = 42, EB3DeltaC n = 37 cells from at least three different cultures, p < 0.0001 by one-way ANOVA, post hoc Dunnett’s test, **p < 0.01, ****p < 0.0001. Growth cones per cells, Control EB3 = 1.862 ± 0.1968 VS EB3M = 0.2143 ± 0.0802 or EB3DeltaC = 0.2162 ± 0.07880; Control EB3 n = 29, EB3M n = 42, EB3DeltaC n = 37 cells from at least three different cultures, p < 0.0001 by one-way ANOVA, post hoc Dunnett’s test ****p < 0.0001, **p < 0.01; Mean ± SEM. Scale bar: 5 μm (**a**) and 10 μm (**d**).

To test this hypothesis, we co-transfected rat hippocampal neurons with Drebrin siRNA - previously shown to specifically knockdown Drebrin in cultured neurons^25^-, or control siRNA along with Lifeact-GFP. 24 hours after plating, we performed time-lapse imaging to analyze F-actin dynamics in stage 2 cells (5 min, imaging every 2 sec). Drebrin siRNA but not control siRNA transfected neurons bear collapsed growth cones with an expected decrease in F-actin treadmilling rate (Fig. 6a–c; Video 8). Drebrin immunostaining confirmed efficient knockdown in Drebrin siRNA transfected neurons whereas control siRNA cells were positive for Drebrin antibody staining (Fig. 6a). To further test the involvement of EB3, we over-expressed different EB3 mutants together with Lifeact-GFP in cultured hippocampal neurons. 24 hrs after plating, we performed time-lapse imaging of stage 2 cells (5 min, imaging every 2 sec). Expression of an EB3 mutant, EB3M, which is capable of binding Drebrin but not microtubules^25^, decreased the F-actin treadmilling speed in neurite tips compared to control cells (Fig. 6d,e; Video 9). Similar results were obtained with another EB3 mutant, EB3DeltaC^25^, which binds microtubule but not Drebrin. EB3DeltaC over-expression decreased F-actin treadmilling in neurite tips (Fig. 6d,e; Video 9). Overall the aforementioned treatments not only decreased F-actin treadmilling but also induced growth cone collapse (Fig. 6c,f) and reduced the number of neurites (Fig. 6c,f). This suggests that Drebrin and EB3 mediated microtubule and actin interaction is essential for growth cone formation, and ultimately for neuronal development.

**Figure 7:**
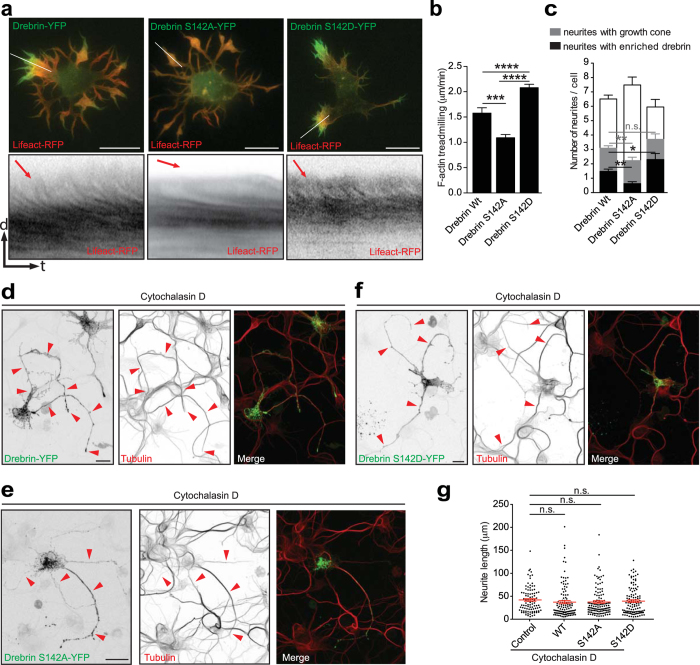
Drebrin phospho-dead and phospho-mimetic mutants overexpression affects F-actin dynamics and growth cone formation. (**a**) Expression of Drebrin phospho-dead mutant (DrebrinS142A) decreased F-actin dynamics and Drebrin phospho-mimetic mutant (DrebrinS142D) increased F-actin dynamics in growth cones (kymographs from white lines). (**b**) Quantification of F-actin treadmilling in growth cones of cells expressing Drebrin, DrebrinS142A and DrebrinS142D. Drebrin = 1.579 ± 0.1030 μm/min; n = 12 cells from at least three different cultures; DrebrinS142A = 1.090 ± 0.0659 μm/min; n = 10 cells from at least three different cultures; DrebrinS142D = 2.08 ± 0.0672 μm/min; n = 10 cells p < 0.0001 by two-way ANOVA, *post hoc* Tukey’s test ***p < 0.001, ****p < 0.0001; Mean ± SEM. (**c**) Quantification of total neurites, neurites with growth cones (gc) and growth cones with enriched Drebrin from stage 2 and 3 cells transfected with Drebrin-YFP, DrebrinS142A-YFP, and DrebrinS142D-YFP were shown. Separate statistical comparisons were made to analyze the differences in the number of neurites with growth cone (black + grey bars) and the number of growth cones with enriched Drebrin (black bars) among the groups. Number of Drebrin-enriched growth cones per cell: Drebrin WT = 1.469 ± 0.1626; DrebrinS142D = 2.300 ± 0.4236; DrebrinS142A = 0.6216 ± 0.1362; Drebrin WT n = 49, DrebrinS142D n = 20, DrebrinS142A n = 37 cells from at least three different cultures, p < 0.0001 by one-way ANOVA, post hoc Dunnett’s test, *p < 0.05, **p < 0.01. Total number of neurite (white + black + grey bars): Drebrin WT = 6.551 ± 0.2124 VS DrebrinS142A = 7.486 ± 0.5164 or DrebrinS142D = 5.950 ± 0.4070, Drebrin WT n = 49, DrebrinS142A n = 37, DrebrinS142D n = 20 cells from at least three different cultures, p = 0.0366 by one-way ANOVA, post hoc Dunnett’s test. Number of neurites with growth cone per cell: Drebrin WT = 3.122 ± 0.1812 VS DrebrinS142A = 2.216 ± 0.2424, Drebrin WT n = 49, DrebrinS142A n = 37 cells from at least three different cultures, p = 0.0004 by one-way ANOVA, post hoc Dunnett’s test, **p < 0.01. Mean ± SEM. (**d**–**e**) Confocal images of rat hippocampal cells transfected with Drebrin WT-YFP (**d**), DrebrinS142A-YFP (**e**), and DrebrinS142D-YFP (**f**) were treated with 5 µM Cytochalasin D at DIV2 and fixed with 4% PFA at DIV3 and stained with α-tubulin antibody (shown in red). (**g**) Quantification of neurite length (µm) after Cytochalasin D treatment. Untransfected control = 42.09 ± 2.813, Drebrin WT = 37.18 ± 3.350, DrebrinS142A = 37.55 ± 2.739, DrebrinS142D = 39.57 ± 2.696, Untransfected control n = 20, Drebrin WT n = 15, DrebrinS142A n = 15, Drebrin S142D n = 20 cells, P = 0.6466 by one-way ANOVA, post hoc Dunnett’s test. Mean ± SEM. Scale bar: 10 μm (**a,d,e** and **f**).

Next, we employed a Drebrin phospho-dead mutant (DrebrinS142A; which inhibits F-actin bundling activity of Drebrin and fails to bind EB3) as well as a Drebrin phospho-mimetic mutant (DrebrinS142D; with an enhanced EB3 binding property^30^). Drebrin contains two F-actin binding domains adjacent to each other, which act cooperatively to bundle F-actin. Phosphorylation of Drebrin at S142 induces an open conformation exposing the two F-actin-binding domains and thereby promoting F-actin bundling^30^. In the closed conformation (i.e., non-phosphorylated state or in the case of phospho-dead mutation) Drebrin binds F-actin but the F-actin bundling activity is repressed together with the binding to EB3. In contrast, in the open conformation (i.e., phosphorylated state or in the case of the phospho-mimetic mutation) Drebrin can bundle F-actin filaments, or straddle existing F-actin bundles and can also bind to EB3^30^. Interestingly, we observed that over-expression of DrebrinS142A-YFP decreased the F-actin treadmilling in growth cones, whereas, DrebrinS142D-YFP overexpression increased the F-actin treadmilling in growth cones compared to Drebrin-YFP expressing cells (Fig. 7a,b; Video 10). Given that our initial findings suggest that accumulation of Drebrin in growth cones correlates with increased F-actin treadmilling, it might be possible that the effect of phospho-mimetic Drebrin is due to an increased number of growth cones per cell enriched with Drebrin. Accordingly, we found that the number of growth cones enriched with Drebrin increased when cells expressed DrebrinS142D-YFP compared to cells expressing Drebrin-YFP (Fig. 7c). On the other hand, DrebrinS142A-YFP, unlike the Drebrin-YFP, failed to have a preferential segregation of Drebrin signal to specific growth cones (Fig. 7c). Moreover, DrebrinS142A-YFP decreased the number of growth cones per cell compared with Drebrin-YFP expressing cells (Fig. 7c). However, the total number of neurites per cell in both mutant conditions did not significantly differ from cells expressing Drebrin-YFP (Fig. 7c). Importantly, we corroborated that the expression of DrebrinS142A-YFP or DrebrinS142D-YFP does not interfere with microtubule polymerization given that F-actin disruption with cytochalasin D did not change the outgrowth of neurites compared to treated control cells or cells expressing Drebrin-YFP (Fig. 7d–g).

**Figure 8:**
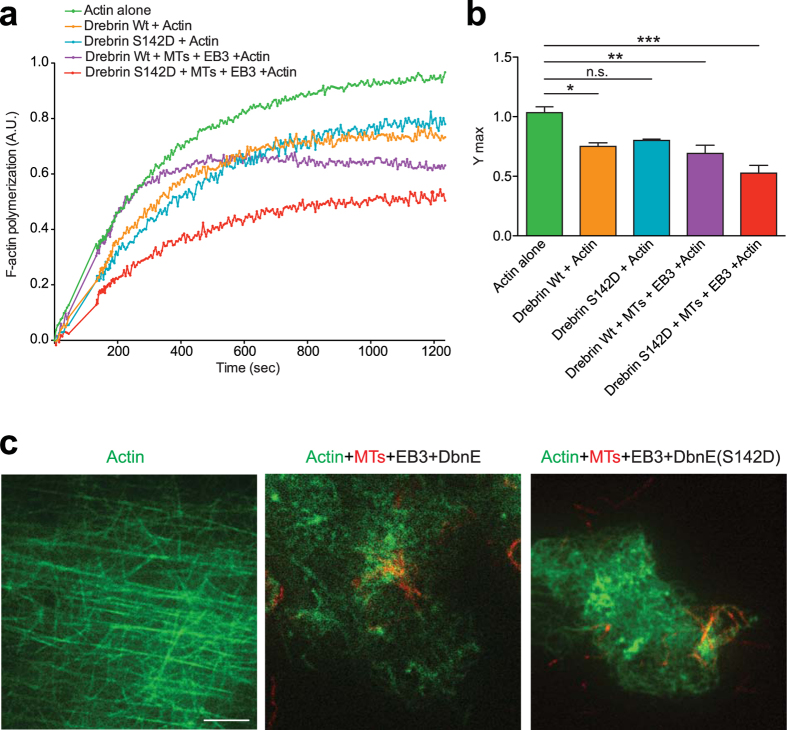
Microtubule/Drebrin interaction affects F-actin polymerization *in vitro*. (**a**) Kinetics of *in vitro* reconstitution assay of actin alone and in the presence of, actin with Drebrin or DrebrinS142D and actin, microtubules, EB3, Drebrin or Drebrin S142D. Curves represent mean values obtained from 2–5 independent experiments. (**b**) Ymax values are calculated from 2–5 independent experiments. Y max values for Actin alone = 1.035 ± 0.04785 A.U.; Actin + Dbn = 0.7504 ± 0.03016; Actin + DbnS142D = 0.8003 ± 0.0116 A.U; Actin + MTs + EB3 + Dbn = 0.6915 ± 0.06875 A.U. Actin + MTs + EB3 + DbnS142D = 0.5267 ± 0.06545 A.U.; *p < 0.05, **p < 0.01, ***p < 0.001 by one-way ANOVA, *post hoc* Dunnett’s test; Mean ± SEM. (**c**) Representative TIRF images of *in vitro* actin polymerization assay under specified conditions. F-actin (labeled by *Alexa-Fluor* 488 phalloidin) is shown in green and microtubules (MT, labeled by HyLight647) are shown in red. Scale bar: 10 µm.

To test more directly whether Drebrin-mediated coupling of microtubules and F-actin influences G-actin polymerization, we performed *in vitro* pyrene actin polymerization assays. Since we used recombinant Drebrin protein purified from bacteria for the *in vitro* assay, the critical Serine 142 phosphorylation of Drebrin (which is necessary for microtubule-F-actin interaction) cannot happen; hence we included the Drebrin phospho-mimetic mutant (DrebrinS142D) in the assay. We found that in the presence of Drebrin or DrebrinS142D together with microtubules and EB3, spontaneous polymerization of actin was decreased compared to control conditions (actin alone). The presence of phospho-mimetic DrebrinS142D mutant, microtubules (MTs) and EB3 further decreased polymerized actin or Ymax (Fig. 8a,b). However, the *t*_*1/2*_ was not significantly changed (*t*_*1/2*_ values for Actin alone = 302.2 ± 106.9 sec; Actin + Dbn = 233.2 ± 57.24 sec; Actin + DbnS142D = 266.7 ± 48.95 sec; Actin + MTs + EB3 + Dbn = 167.2 ± 61.07 sec. Actin + MTs + EB3 + DbnS142D = 236.3 ± 101.2 sec; n = 2–5; p = 0.799 by one-way ANOVA, *post hoc* Dunnett’s test). Importantly, by TIRF microscopy of *in vitro* reconstitution assays we observed that the structures of spontaneously polymerized F-actin, which appeared as a network or lattice of filaments, are disrupted in the presence of Drebrin or DrebrinS142D along with microtubules and EB3 (Fig. 8c) and F-actin clouds were predominant under these conditions. Thus, microtubule dynamics directly affect actin polymerization via Drebrin and EB3.

## Discussion

Neuronal polarization is a complex process, which starts with breaking cellular symmetry and eventually determines the morphological and functional orientation of a neuron to allow for a correct information flow in a given neuronal network. The crucial initial step in this tightly regulated process is axon selection. The position of the centrosome has been suggested as a landmark to predict the position of axon outgrowth^2, 17–19^. Importantly, we now provide a mechanistic link between the microtubule network and F-actin dynamics prior and during initial axon formation. More specifically we could demonstrate in this study that i) modulation of F-actin dynamics by microtubules affects growth cone behavior and ultimately axon formation, ii) Drebrin is a molecular linker between microtubules and F-actin in this process and finally iii) disturbance of this molecular pathway impairs neuronal development.

It is known that during the transition from stage 2 to 3 the growth cone of the neurite, which elongates as an axon, contains more dynamic F-actin^12^ and more stable microtubules^10^. However, it is not clear whether the interplay between F-actin and microtubules leads to this transition. Here we show that fewer or unstable microtubules decrease F-actin dynamics, and more and stable microtubules increases F-actin dynamics. We therefore propose that the microtubule network may have an intrinsic signaling capacity to induce initial polarization as suggested previously in dissociated hippocampal neurons^2, 17^.

Our data implicate Drebrin as a molecular linker, which allows microtubule to modulate F-actin. Drebrin promotes microtubule entry into F-actin-rich structures, such as synaptic spines and growth cones^22, 30^ (and Fig. 1). It has been unclear whether a Drebrin-mediated molecular interplay directly affects F-actin dynamics in growth cones. Here we show that Drebrin (together with more microtubule plus-ends) is segregated to growth cones with faster F-actin treadmilling before and during initial axon formation. This segregation predicts the position of axon formation. Interaction of Drebrin with microtubule allows for more dynamic F-actin. Silencing Drebrin or disruption of the Drebrin-microtubule interaction prominently affects F-actin dynamics and in consequence neuronal development. Importantly, given that Drebrin over-expression reduced overall F-actin dynamics due to its reported stabilizing role^24^, we propose that axon formation might take place at the expense of specific segregation/stabilization of microtubules. In this scenario the external environment, which governs the final axon trajectory^3, 16, 31–34^, could also instruct specific microtubules segregation prior to axon extension. Thus, axon selection may be regulated by intrinsic mechanisms that are influenced by extracellular cues^35–37^. It will be interesting to learn how Drebrin-microtubule-F-actin interaction is coupled to intracellular signaling cascades that are controlled by these external cues.

## Material and Methods

### RNAi and fluorescent protein constructs

F. Bradke (DZNE) kindly provided Lifeact-EGFP and EB3-mCherry. P. Gordon-Weeks kindly provided Drebrin-YFP (Addgene plasmid # 40359)^25^ and DrebrinS142A-YFP (Addgene plasmid # 58335)^30^. M. Davidson kindly provided Lifeact-RFP (Addgene plasmid # 54586). M. Kneussel (ZMNH, UKE) kindly provided EB3-GFP. For in utero electroporation experiments, Cep120 shRNA construct, Cep120 i2968 - published earlier by us in Xie *et al*., 2007 -was used and a pSilencer vector containing a random sequence hairpin insert was employed as a control-shRNA. The plasmid constructs expressing EB3M and EB3DeltaC (truncated forms of EB3) were generated as described earlier^25^. EB3M and EB3DeltaC sequences were amplified by polymerase chain reaction (PCR) from the EB3-mCherry construct using forward and reverse primers containing suitable restriction sites for cloning into the pmCherry-C1vector (Clontech). EB3M forward primer: 5′-CTTAATGGATCCCGGCAGGGCCAGGACGTAG-3, EB3M reverse primer: 5′-CTGGCTGAATTCGATGCCTGAGATAACA-3′, EB3DeltaC forward primer: 5´-CTGGCTGGATCCATGGCCGTCAATGTGTACTCC-3′, EB3DeltaC reverse primer: 5´- CTGGCTGAATTCGCCGCCATTTCGGGCTGATGG -3′. PCR products of EB3M and EB3DeltaC were digested with BamHI and EcoRI and inserted into the pmCherry-C1 vector (Clontech) digested with BglII and EcoRI. The pmCherry-EB3M and pmCherry-EB3DeltaC plasmid constructs were thus generated and verified by DNA sequencing. siRNA duplexes against rat Drebrin (5′-GAGAACCAGAAAGUGAUGUACdTdT-3′ (sense) and 3′-dTdTCUCUUGGUCUUUCACUACAUG-5′ (antisense)) (previously published by^25^ and control siRNA (ON-TARGETplus Non-targeting Pool) were synthesized by Dharmacon.

### Animal experiments

Animal (rat and mouse) experiments were performed according to the German and European Animal Welfare Act and with the approval of local authorities of the city-state Hamburg (Behörde für Gesundheit und Verbraucherschutz, Fachbereich Veterinärwesen; G48/13) and the animal care committee of the University Medical Center Hamburg-Eppendorf.

### In utero electroporation

Pregnant C57BL/6 mice with E15 embryos were first administered with pre-operative analgesic, Buprenorphine (0.1 mg/kg), by subcutaneous injection. 30 min later the mice were anaesthetized with isoflurane (4% for induction, 2–3% for maintenance) in oxygen (0.5–0.8 L/min for induction and maintenance). Later, the uterine horns were exposed and plasmids mixed with Fast Green (Sigma) were microinjected into the lateral ventricles of embryos. The concentration of shRNA (control, Cep120-shRNA) was 3-fold higher than that of Lifeact-GFP. Five current pulses (50 ms pulse / 950 ms interval; 35–36 V) were delivered across the head of the embryos. After surgery, mice were kept in a warm environment and provided with moist food containing post-operative analgesic, Meloxicam (0.2–1 mg/kg), until they were euthanized for collecting the brains from the embryos.

### Preparation of hippocampi from rat and cortices from mouse embryos

Pregnant rats and mice were anaesthetized with CO_2_/O_2_ and then euthanized before taking the embryos out from their uteruses. Embryos were then decapitated and heads were collected in petri dishes kept on ice. After opening the skulls, brains were collected in fresh petri dishes with HBSS on ice. Hemispheres were separated, meninges were carefully stripped away and hippocampi or cortices were dissected under a dissecting microscope.

### Cortical neuron cultures

Neurons were transfected by in utero electroporation at E15 and transfected cortices were isolated (as explained above) two days later. Isolated cortices were triturated in 1xHBSS (Invitrogen) containing papain and DNase at 37 °C (Worthington). Neurons were plated on poly-L-lysine coated glass coverslips in Neurobasal/B27 medium (Invitrogen), maintained in culture for 24 hours for time-lapse imaging.

### Hippocampal neuronal cultures and transfections

Isolated hippocampi (from E18 embryos) were triturated in 1xHBSS (Invitrogen) after digestion by papain and DNase for 10 min at 37 °C (Worthington). Transfections were performed using the Amaxa nucleoporation system following the manufacturer’s manual. For each transfection 5 × 10^6^ cells and 5 µg of DNA mix were used for transfecting either control siRNA or Drebrin siRNA, and 3 µg of DNA mix for each of the remaining transfections. After electroporation, neurons were plated on poly-L-lysine coated glass coverslips (for immunostaining), on glass-bottomed dishes (ibidi, for live imaging) or tissue culture chambers (Sarstedt, for live imaging) in Neurobasal/B27 medium (Invitrogen), maintained in culture for 4~48 hours at 37 °C with 5% CO_2_ before use.

### Pharmacological treatments

Nocodazole (a microtubule-destabilizing drug) was used at a final concentration of 7 µM. Taxol, a microtubule-stabilizing drug, was used at a final concentration of 10 nM. Cytochalasin D, (an inhibitor of actin polymerization as well as a depolymerizer of actin filaments) was used at a final concentration of 5 µM. All the compounds were purchased from Sigma.

### Immunocytochemistry

Rat hippocampal neurons grown on coverslips were fixed with 4% paraformaldehyde (PFA) at 37 °C for 10 min and then permeabilized with 0.5% triton-x 100 for 10 min. Nonspecific binding was blocked by incubation in PBS with 5% donkey serum, 2% goat serum and 1% BSA for 60 min at RT, followed by specific primary antibody incubation: mouse anti-Drebrin (Abcam, ab12350), rabbit anti-cofilin (abcam ab42824), mouse anti-α-tubulin (Abcam, ab7291) or mouse anti-Tau1 (Millipore, MAB3420) was added for incubation for 120 min at RT. The respective anti-mouse anti-rabbit Alexa fluor -488, -568 or -647 labeled secondary antibody was added for 60 min at room temperature. Primary and secondary antibodies were diluted in PBS with 2.5% donkey serum, 1% goat serum and 0.5% BSA. After primary as well as secondary antibody incubation three washing steps with PBS were performed. For F-actin labelling Acti-stain 488 phalloidin (Cytoskeleton) was used at 1 to 120 dilution in 1X PBS and incubated for 30 min at RT followed by 3 short PBS rinses. Coverslips were mounted onto the slides using ProLong Gold (Invitrogen) with or without nuclear stain DAPI (Invitrogen) and were stored light-protected.

### Epi-fluorescence imaging

Epi-fluorescence imaging was performed on an inverted Nikon microscope (Eclipse, Ti) with a 60x objective (NA 1.4). During time-lapse imaging, cells plated on a glass-bottomed dish (ibidi) or a culture chamber (Sarstedt) were kept in an acrylic chamber at 37 °C in 5% CO_2_. Light intensity of each channel was normally set at 8, with exposure time of 300–800 ms. Images were captured with a CoolSNAP HQ2camera (Roper Scientific) using NIS-Elements AR software (version 4.20.01 from Nikon Corporation).

### Confocal imaging

Images were taken using a Zeiss LSM 700 confocal laser-scanning microscope with 40X objectives (NA 1.3).

### TIRFM imaging

TIRF microscopy for *in vitro* actin and MT polymerization assays were performed on a Visitron Systems VisiScope TIRF/FRAP imaging system based on Nikon Ti-E equipped with a perfect focus system (Nikon), Nikon CFI Apo TIRF 100x, 1.49 N.A. oil objective, a back focal TIRF scanner for suppression of interference fringes (Ilas-2, Roper Scientific France/ PICT-IBiSA, Institut Curie) and controlled with VisiView software. 405, 561, 488 and 647 nm laser lanes were used for illumination and activation of respective fluorophores. Fluorescence was collected through mCherry and ET 405/488/561/640 Laser Quad Band filters with a The ORCA-Flash 4.0 LT sCMOS camera.

### Analysis of F-actin treadmilling in growth cones

For analysis of F-actin treadmilling in growth cones, 5 min time-lapse videos of Lifeact-GFP or Lifeact-RFP expressing rat hippocampal or mouse cortical neurons were used. Kymographs were generated from transfected growth cones using ImageJ^38–40^. From the kymographs (generated by setting line width to 1), the slopes of retrograde F-actin trajectories were measured and average slope was represented in µm per min.

### EB3 comets quantifications

The number of EB3 comets entering each neurite was measured from the time-lapses of stage 2 hippocampal neurons transfected with EB3-mCherry and Lifeact-GFP. Lines were drawn along the width of each neurite at the neck of the growth cones of stage 2 neurons, to generate kymographs (with a line width of 1). From the kymographs, the number of EB3 comets (that appear as distinct spots) was measured.

### Drebrin and Cofilin fluorescence intensity measurement in growth cones

Fluorescence intensities of endogenous Drebrin (stained by Drebrin antibody), Drebrin overexpressing neurons (the 5 min time-lapse videos of cells transfected with Drebrin-YFP plasmid) and endogenous cofilin (stained by cofilin antibody) in all the growth cones of the neurites from stage 2 cells were measured.

For Drebrin and/or cofilin intensity measurements in the growth cones (in Figs 1E, 2C and Supplementary Figure 1H), we delineated the individual growth cone areas for each neurite manually and measured the mean intensities using Image J.

In case of Drebrin-YFP overexpression time-lapse videos (in Fig. 2E), for delineation, each neurite growth cone area was cropped from the 5 min time-lapse video and mean auto thresholding method (Image J) was applied. Using this approach, we were able to define the location of the dynamic growth cones more precisely throughout the time-lapse from all the 151 frames of 5 min videos (frame interval, approx. 2 sec). We used the obtained xy coordinates to retrieve the original intensity values from the growth cones. Since there are 151 frames in the time lapses of Drebrin overexpressing cells, the procedure was automated with a script in R (R Development Core Team (2008). R: A language and environment for statistical computing. R Foundation for Statistical Computing, Vienna, Austria)

### Measurement of percentage of EB3 comet coverage

5 min time-lapse videos from cells co-transfected with either Lifeact and EB3 or Drebrin and EB3 were selected. The max-projection of each time-lapse was created under ImageJ. Area covered by Lifeact, Drebrin or EB3 was measured respectively in growth cones. The percentage of Lifeact- or Drebrin- covered area occupied by EB3 signal was quantified.

### Bacterial Expression of Drebrin E

The cDNA of Drebrin E was cloned into the vector *pGEX-6P-2A encoding* an *N-*terminal glutathione S-transferase (GST) tag. The phospho-mimetic mutant of Drebrin (DrebrinS142D) was generated from the cDNA of Drebrin E by Quick change PCR. The Drebrin and DrebrinS142D plasmids were transformed into E. coli BL21 pLysRep. After the cells were grown to an OD of 0.6, expression of the GST fusion proteins were induced by adding 0.2 mM Isopropyl-β-D-thiogalactopyranoside (BioTechTrade & Service GmbH), and incubation was continued for 20 hours at 10 °C. GST-Drebrin and GST-DrebrinS142D were purified from lysed bacteria by glutathione-affinity-chromatography. Protein concentration was determined by Coomassie-stained SDS-PAGEs using BSA as standard.

### TIRF MT-actin assays

G-actin (1 mg/ml) *Cytostebu-bio*, *Cytoskeleton*) was incubated on ice for 1 hour and centrifuged at 4 °C for 20 min at 14000 g. HiLyteFluor 647-tubulin was purchased from Cytoskeleton and HyLight647-MT seeds were made as described in Mohan *et al*. 2013^41^ and stored at −80 °C. For the experiment seeds were quickly transferred into a 37 °C water bath, incubated for 20 min and kept in the dark at RT for 1–2 hrs. Labeled MTs were diluted 1:40 in PEM80 (80 mM PIPES, pH 6.9, 2 mM MgCl_2_, 1 mM EGTA) containing 10 µM of taxol (Sigma). 8 µl Drebrin or DrebrinS142D (50 ng/µl), 1 µl EB3 (100 ng/µl), 1 µl ATP (5 mM), 1 µl GTP (20 mM), 2 µl taxol (200 µM), 2 µl 10x GT-buffer (800 mM PIPES pH 7.0, 20 mM MgCl_2_, 5 mM EGTA), 2 µl Alexa-Fluor®488 labeled phalloidin (1:25 dilution), 2 µl G-actin (1 mg/ml; tebu-bio, Cytoskeleton), 1 µl MTs were added to a microtube, mixed, applied onto a poly-L-lysine covered chamber slide (Ibidi, Munich) and analyzed by TIRF-microscopy. TIRFM was performed on Visiscope Imaging system described above.

### Pyrene actin polymerization assays

G-actin (1 mg/ml; *tebu-bio, Cytoskeleton*) and pyrene-labelled G-actin (0.1 mg/ml; *tebu-bio, Cytoskeleton*) were incubated on ice for 1 hour and centrifuged at 4 °C for 20 min at 14000 g. To analyse actin polymerization, 40 µl Drebrin wt or DrebrinS142D (50 ng/µl), 5 µl EB3 (100 ng/µl), 5 µl ATP (5 mM), 5 µl GTP (20 mM), 10 µl taxol (200 µM), 10 µl MTs prepared as indicated earlier, 5 µl G-actin (1 mg/µl) and 10 µl pyrene labelled G-actin (0.1 mg/ml) were added to microtube, mixed and transferred to a 96-well plate. Pyrene fluorescence was monitored via TecanSaphire 2 reader at excitation level of 365 nm with Tecani-control 1.5 and after ten cycles 10x *polymerization* buffer (50 mM Tris-HCl, pH 7.5, 500 mM KCl, 20 mM MgCl_2_, 10 mM EGTA, 2 mM ATP) was added to start polymerization. Subsequently pyrene fluorescence was monitored for further 30 min.

### Image processing

Linear adjustments of brightness and contrast were performed on images using Photoshop CS or ImageJ.

### Statistical analysis

Statistical analysis was performed using the GraphPad Prism 6 software. Data shown in the graphs were collected from at least three independent experiments. The Student’s t test (two-tailed) was used to compare means of two groups, whereas analysis of variance (ANOVA) test was used when comparing more than two groups. Asterisks *, **, *** and ****represents p < 0.05, 0.01, 0.001 and 0.0001 respectively. Error bars in the graphs always represent standard error of mean. For Correlation analysis, values were normalized according to standard score. Mean values from each group were tested for normality using D’Agonstino & Pearson normality test. All groups passed this test. Linear regression was performed while testing for linearity as well as a significant deviation of the slope from non-zero. In case the deviation from linearity was significant, a non-linear second order polynomial fit was carried out. The polynomial fit was then tested for deviation of the data from the model and appeared to be non-significant. Pearson correlation analysis was performed for showing the linear relationship between two sets of data.

## Electronic supplementary material

Supplementary Information

Video 1

Video 2

Video 3

Video 4

Video 5

Video 6

Video 7

Video 8

Video 9

Video 10
