# Supplementary material for: Microtubules Modulate F-actin Dynamics during Neuronal Polarization
Source: Sci Rep. 2017 Aug 29;7:9583. doi: 10.1038/s41598-017-09832-8 (PMC5575062; doi:10.1038/s41598-017-09832-8)

Supplementary information

Microtubules Modulates F-actin Dynamics during Neuronal Polarization

Bing Zhao[1§], Durga Praveen Meka[1§], Robin Scharrenberg[1], Theresa König[1], Birgit Schwanke[1], Oliver Kobler[6], Sabine Windhorst[5], Michael R. Kreutz[3,4], Marina Mikhaylova[2], Froylan Calderon de Anda[1*]

[1]RG Neuronal Development, Center for Molecular Neurobiology Hamburg (ZMNH), University Medical Center Hamburg-Eppendorf, 20251 Hamburg, Germany.

[2]Emmy-Noether Group “Neuronal Protein Transport”, Center for Molecular Neurobiology (ZMNH), University Medical Center Hamburg-Eppendorf, 20251 Hamburg, Germany.

[3]RG Neuroplasticity, Leibniz Institute for Neurobiology, 39118 Magdeburg, Germany

[4]Leibniz Guest Group “Dendritic Organelles and Synaptic Function”, Center for Molecular Neurobiology (ZMNH), University Medical Center Hamburg-Eppendorf, 20251 Hamburg, Germany.

[5]Department of Biochemistry and Signal Transduction, University Medical Center Hamburg-Eppendorf, 20246 Hamburg, Germany.

[6]Combinatorial Neuroimaging Core Facility (CNI), Leibniz Institute for Neurobiology, 39118 Magdeburg, Germany.

[§] Equal contribution

To whom correspondence should be addressed:

Froylan Calderon de Anda
Email: froylan.calderon@zmnh.uni-hamburg.de
(O): +49 40 7410-56817
(F): +49 40 7410-56450

Supplementary Figure 1. Endogenous cofilin colocalize with endogenous and overexpressed drebrin in growth cone. (**a**) Endogenous drebrin localized in the central and peripheral domain of growth cones. Stage 2 neuron labeled with phalloidin (F-actin, red), drebrin antibody (green), and DAPI (blue). Drebrin is enriched in the central domain of some growth cones, although is not precluded from the peripheral domain. (**b**) Drebrin and F-actin signal profile from growth cones from panel a (white line). (**c**) Endogenous cofilin colocalized with endogenous drebrin. Neurons were labeled with cofilin antibody (red), drebrin antibody (green), and DAPI (blue). (**d**) Cofilin and Drebrin signal profile from growth cones from panel c (white line). (**e**) Endogenous cofilin colocalized with overexpressed drebrin. Neurons were transfected with Drebrin-YFP (green) and then labeled with cofilin antibody (red) and DAPI (blue). (**f**) Cofilin and overexpressed Drebrin signal profile from growth cones from panel e (white line). (**g**) Left column, endogenous drebrin and cofilin distribution in Stage 2 neuron. Right column, drebrin and endogenous cofilin distribution in drebrin-overexpressed neuron. (**h**) Correlation of cofilin intensity with the intensity of endogenous and overexpressed drebrin. For endogenous drebrin correlation, n = 9 cells from at least three different cultures.Values were normalized according to standard score and axes are represented in unit of standard deviation [σ]. Line equation: Y = 0.8040*X - 3.928e-008; Pearson r = 0.804, p<0.0001. Dashed lines represent 95% confidence intervals. . For overexpressed drebrin correlation, n = 10 cells from at least three different cultures.Values were normalized according to standard score and axes are represented in unit of standard deviation [σ]. Line equation: Y = 0.9029*X + 2.084e-008; Pearson r = 0.9029, p<0.0001. Dashed lines represent 95% confidence intervals. Scale bar: 10 µm (a, c, e and g)

Supplementary Figure 2. Axons of drebrin overexpressing cells have relatively higher F-actin dynamics irrespective of the drebrin-induced stabilization of F-actin in stage 1, stage 2 and stage 3 cell neurites. Quantification of F-actin

treadmilling in neurites from stage 1, stage 2 and stage 3 cells and stage 3 axons expressing Lifeact-GFP alone and Lifeact-RFP together with drebrin-YFP. Lifeact-GFP stage 1 = 4.327 ± 0.1601 μm/min from n = 20 cells; Lifeact-RFP + drebrin-YFP stage 1 =1.903 ± 0.1143μm/min; n = 9 cells from at least three different cultures; $p<0.0001$ by one-way ANOVA, post hoc Bonferroni's test ****$p<0.0001$. Lifeact-GFP stage 2 = 5.130 ± 0.0.1017 μm/min from n = 15 cells; Lifeact-RFP + drebrin-YFP stage 2 = 1.903 ± 0.1143μm/min; n = 10 cells from at least three different cultures; $p<0.0001$ by one-way ANOVA, post hoc Bonferroni's test ****$p<0.0001$. Lifeact-GFP stage 3 = 4.553 ± 0.1088 μm/min from n = 12 cells. Lifeact-RFP + drebrin-YFP stage 3 =1.162 ± 0.2397 μm/min; n = 6 cells from at least three different cultures; $p<0.0001$ by one-way ANOVA, post hoc Bonferroni's test ****$p<0.0001$. Lifeact-GFP stage 3 axon = 5.448 ± 0.1770 μm/min from n = 12 cells; Lifeact-RFP + drebrin-YFP stage 3 axon = 3.269 ± 0.6442 μm/min; n = 6 cells from at least three different cultures; $p<0.0001$ by one-way ANOVA, post hoc Bonferroni's test ***$p<0.001$; Lifeact-RFP + drebrin-YFP stage 1 =1.903 ± 0.1143μm/min; n = 9 cells from at least three different cultures; Lifeact-RFP + drebrin-YFP stage 2 = 1.903 ± 0.1143μm/min; n = 10 cells from at least three different cultures; Lifeact-RFP + drebrin-YFP stage 3 =1.162 ± 0.2397 μm/min; n = 6 cells from at least three different cultures, Lifeact-RFP + drebrin-YFP stage 3 axon = 3.269 ± 0.6442 μm/min; n = 6 cells from at least three different cultures; $p<0.0001$ by one-way ANOVA, post hoc Bonferroni's test **$p<0.01$, ***$p<0.001$, ****$p<0.0001$; Mean ± SEM.

Video 1. Time-lapse imaging of growth cones expressing drebrin-YFP/EB3-mCherry (left video) and Lifeact-GFP/EB3-mCherry (right video). Epi-fluorescence imaging was performed on an inverted Nikon microscope (Eclipse, Ti) with a 60x objective (NA 1.4). Duration of time-lapse imaging: 5 min acquiring images every 2 sec.

Video 2. Time-lapse imaging of stage 2 neuron expressing EB3-mCherry and Lifeact-GFP. Epi-fluorescence imaging was performed on an inverted Nikon microscope (Eclipse, Ti) with a 60x objective (NA 1.4). Duration of time-lapse imaging: 5 min acquiring images every 2 sec. After the time-lapse, cell was fixed and stained for drebrin.

Video 3. Time-lapse imaging of early stage 3 neuron expressing drebrin-YFP and Lifeact-RFP. Epi-fluorescence imaging was performed on an inverted Nikon microscope (Eclipse, Ti) with a 60x objective (NA 1.4). Duration of time-lapse imaging: 5 min acquiring images every 2 sec.

Video 4. Long-term time-lapse imaging of a stage 2 neuron expressing drebrin-YFP and Lifeact-RFP. Epi-fluorescence imaging was performed on an inverted Nikon microscope (Eclipse, Ti) with a 60x objective (NA 1.4). After the time-lapse, cell was fixed and stained for the axonal marker Tau-1. Duration of time-lapse imaging: 12 hr acquiring images every 5 min. After the time-lapse, cell was fixed and stained for Tau-1.

Video 5. Time-lapse imaging of stage 2 and early stage 3 neurons expressing EB3-mCherry and Lifeact-GFP. Epi-fluorescence imaging was performed on an inverted Nikon microscope (Eclipse, Ti) with a 60x objective (NA 1.4). Duration of time-lapse imaging: 5 min acquiring images every 2 sec.

Video 6. Time-lapse imaging of stage 2 neurons expressing EB3-mCherry and Lifeact-GFP. Cell was imaged before nocodazole treatment for 5 min (acquiring images every 2 sec). After nocodazole treatment the same cell was imaged 3 times more (20 min, 95 min, and 120 min) for 5 min (acquiring images every 2 sec) each

time. Epi-fluorescence imaging was performed on an inverted Nikon microscope (Eclipse, Ti) with a 60x objective (NA 1.4).

Video 7. Time-lapse imaging of stage 2 neuron expressing EB3-mCherry and Lifeact-GFP. Cell was imaged before taxol treatment for 5 min (acquiring images every 2 sec). After taxol treatment the same cell was imaged 2 times more (2.5 hr and 4 hr) for 5 min (acquiring images every 2 sec) each time. Epi-fluorescence imaging was performed on an inverted Nikon microscope (Eclipse, Ti) with a 60x objective (NA 1.4).

Video 8. Time-lapse imaging of stage 2 neuron transfected with Lifeact-GFP together with drebrin siRNA. Epi-fluorescence imaging was performed on an inverted Nikon microscope (Eclipse, Ti) with a 60x objective (NA 1.4). Duration of time-lapse imaging: 5 min acquiring images every 2 sec.

Video 9. Time-lapse imaging of stage 2 neurons expressing control (mCherry-EB3), mCherry-EB3M, and mCherry-EB3DeltaC together with Lifeact-GFP. Epi-fluorescence imaging was performed on an inverted Nikon microscope (Eclipse, Ti) with a 60x objective (NA 1.4). Duration of time-lapse imaging: 5 min acquiring images every 2 sec.

Video 10. Time-lapse imaging of stage 2 neurons expressing drebrin-YFP and drebrin S142A-YFP or drebrin S142D-YFP together with Lifeact-RFP. Epi-fluorescence imaging was performed on an inverted Nikon microscope (Eclipse, Ti) with a 60x objective (NA 1.4). Duration of time-lapse imaging: 5 min acquiring images every 2 sec.

Supplementary Figure 1

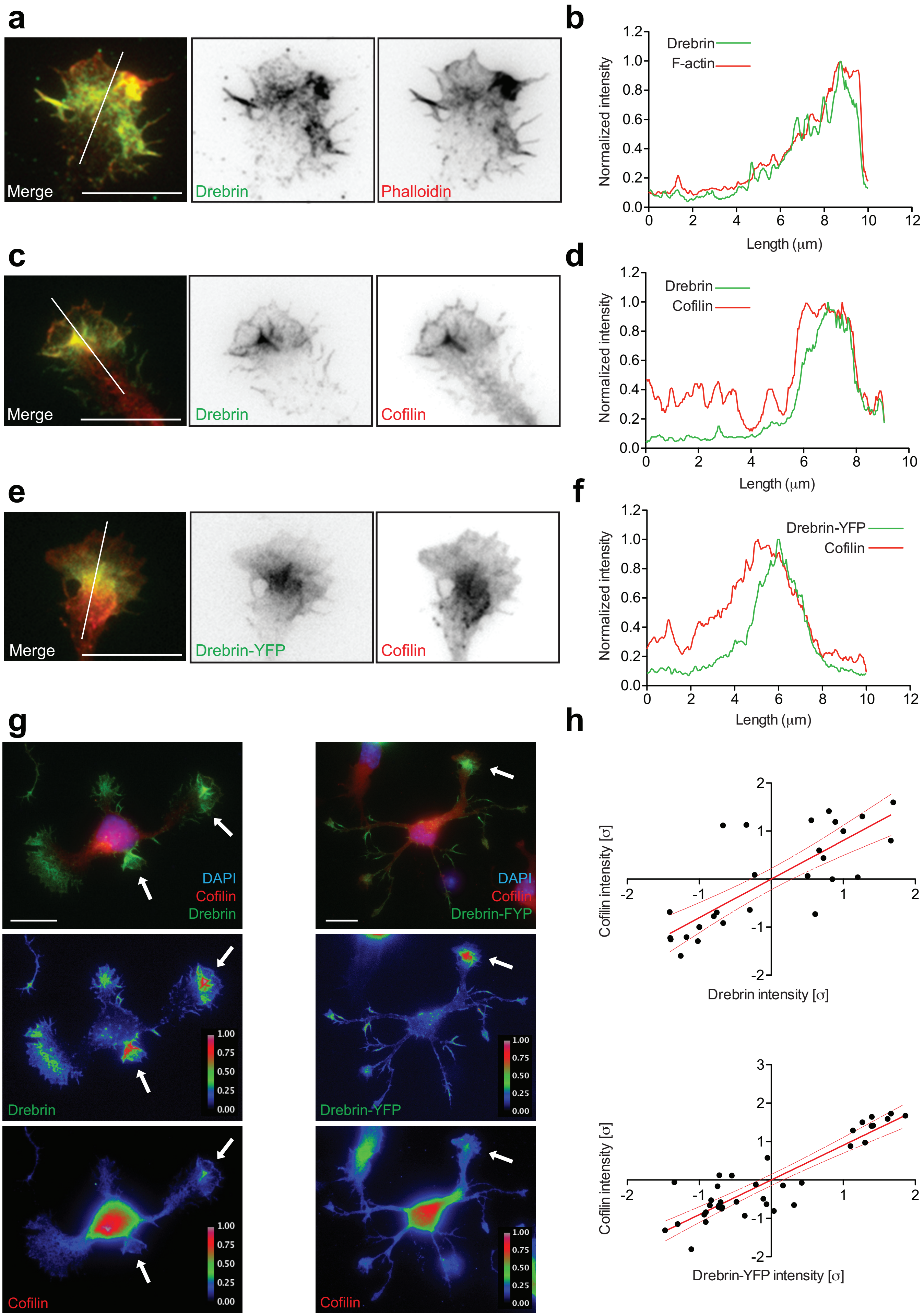

Supplementary Figure 2

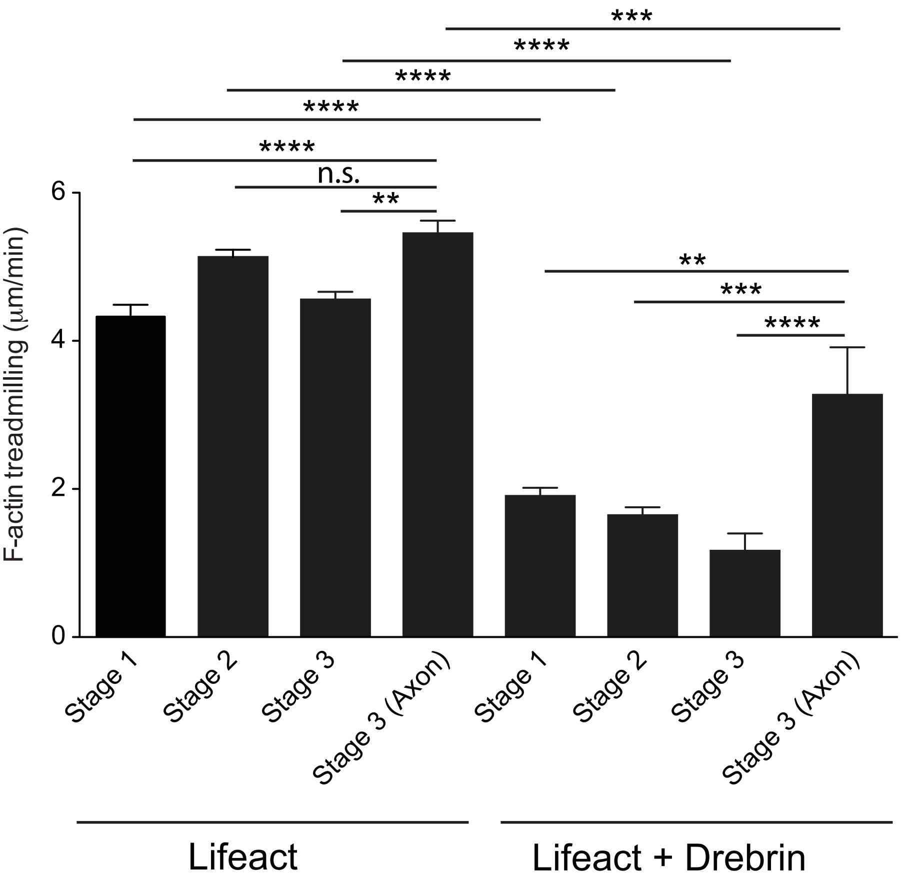

Supplement: Supplementary file 1 — Supplementary Information [file 41598_2017_9832_MOESM1_ESM.pdf]
